# Dr. Allen E. Baker

**Published:** 1916-02

**Authors:** 


### OBITUARY

---

**Readers are requested to report promptly the death of all physicians in Western New York, or former residents of this region, or graduates of any medical school in Western New York, and to notify the families of the deceased of our desire to publish adequate Obituary notices.**

---

**Dr. Allen E. Baker,** N. Y. Homoeopathic 1888, died at his home in Auburn, Dec. 15, 1915, his age being stated at 43, which is not compatible with the date of his graduation.

